# Insights into Neuromuscular Function in Older Adults from Functional Data Analysis of Time-Dependent Handgrip Strength Curves

**DOI:** 10.3390/bioengineering13040381

**Published:** 2026-03-26

**Authors:** Diana Urbano, Mário Inácio, Maria Teresa Restivo

**Affiliations:** 1 LAETA—INEGI, Faculty of Engineering, University of Porto, Rua do Dr. Roberto Frias, 4200-465 Porto, Portugal; 2 University of Maia, Avenida Carlos Oliveira Campos, Castelo da Maia, 4475-690 Maia, Portugal; 3 Research Center in Sport Sciences, Health Sciences and Human Development, University of Trás-os Montes e Alto Douro, Quinta dos Prados, 5000-801 Vila Real, Portugal; 4 Centre for Movement, Occupation and Rehabilitation Sciences, Oxford Brookes University, Hadington Campus, Oxford OX3 0BP, UK; 5 Agency for Assessment and Accreditation of Higher Education (A3ES), Praça de Alvalade, 6-5º Frente, 1069-061 Lisboa, Portugal

**Keywords:** functional data analysis, FPCA, handgrip strength, neuromuscular control, older adults, SnPM

## Abstract

Handgrip strength (*HGS*) is widely used as a biomarker of muscle function and overall health in older adults. However, conventional analyses based on peak force values may overlook relevant temporal features of the *HGS* curve. This cross-sectional study proposes a novel methodological approach that examines the shape and variability of *HGS*(*t*) curves recorded from community-dwelling older adults. Functional principal component analysis (FPCA) was applied to assess the consistency of individual trials and the representativeness of mean curves. Statistical non-parametric mapping (SnPM) was then used to identify time regions showing significant differences between groups. Complementary analyses of discrete and derivative parameters, together with non-parametric comparisons based on the Hodges–Lehmann estimator and corresponding 95% confidence intervals, were conducted to quantify effect sizes. FPCA revealed high within-participant consistency, supporting the use of mean curves for group-level comparisons. SPM analyses indicated significant differences in the early force development phase. Importantly, this approach shows that sex differences are attributable to magnitude effects, with men generating higher forces and faster early rates of force development, and not to differences in the neuromuscular strategy of force production. Traditional discrete parameters partly captured these patterns but failed to reflect the full temporal dynamics. This methodological approach to the *HGS* curve may provide further insights into neuromuscular control mechanisms that cannot be truly captured by the minimalistic *HGS* discrete parameters.

## 1. Introduction

Looking at handgrip strength as a function of time, *HGS*(*t*), adds clinically useful information that the well-established health tracking biomarker, peak *HGS* [1,2,3], cannot give alone. In fact, the first 50–250 milliseconds of force production reflect how quickly the nervous system can recruit and fire motor units and how efficiently force is transmitted through the musculotendinous system [4,5,6,7,8]. Measures, such as rate of force development (*RFD*), and early force levels at 25/50/75 ms may give an indication of those processes. Early-phase *RFD* is especially sensitive to neural factors and aging-related changes in motor unit behavior that may change even if peak force does not [9,10,11,12].

Peak dynamics, i.e., maximum force (*Fmax*), the corresponding time to peak (*tmax*) and the shape around peak, tell how force rises and transitions, providing information that can distinguish between patterns like, for instance, similar peaks but faster rises. *HGS*(*t*) also reveals when and where differences occur (early rise vs. late maintenance), which can also give valuable information for training or rehabilitation [13,14].

Integrals or fatigability indices after peak force (sustained grip) can be used to quantify static endurance, measuring the ability to sustain force [15].

The dynamics of individuals’ grip performance over time not only reflect neuromuscular capabilities but also shed light on broader physiological and functional traits such as mobility, balance and frailty. Comparisons of *HGS*(*t*) descriptors across different populations, by age, functional status, and health condition, have been shown to capture differences in physical function [5,16,17].

In athletic and physical activity groups, studies have demonstrated associations between force–time characteristics, such as *RFD*, and sport-specific performance, as well as overall strength and muscular fatigue resistance [13,18,19].

Among older adults, *HGS*(*t*) metrics have been linked to indicators of functional and daily-life performance, distinguishing between sedentary and active [20,21] and between frail and non-frail individuals [22].

It is therefore accepted that *HGS*(*t*) descriptors likely map onto meaningful physiological or functional divergences by revealing differences not only in maximal strength but also in how quickly force is produced, how well it is sustained, and how susceptible the individual is to muscular fatigue.

While *HGS*(*t*) discrete parameters, such as maximum HGS, *Fmax*, *tmax*, different *RFDs*, fatigue slopes and time curve integrals, are often used as surrogate markers of robust health and function, they have a significant limitation, as they represent global quantities in specific time intervals. Consequently, there is the possibility of missing valuable localized temporal differences, or group divergences across curves, that occur at certain phases of the handgrip force production. Moreover, these descriptors are sensitive to the sampling rates of the devices used to measure *HGS*(*t*) and preprocessing of the *HGS*(*t*) data that can render comparisons across studies problematic [23].

This work presents and explores a function/continuous curve framework to analyze sex differences in *HGS*(*t*) curves within an older adult cohort. The functional analysis pipeline comprises two key steps: functional principal component analysis (FPCA) [24,25] followed by statistical non-parameter mapping (SnPM) [26,27,28].

FPCA has been used in biomechanics and movement science to characterize gait kinematics and kinetics [29], analyze complex multidimensional movement patterns [30], and describe variation in electromyography (EMG) profiles [31].

SPM has become a standard method for statistically comparing entire biomechanical or physiological waveforms without reducing them to discrete points. It has been used to evaluate differences in kinematic force trajectories, EMG curves, and neuromuscular and physiological time series patterns [28,32,33,34]. The non-parametric version of SPM (SnPM) is employed in the case of non-normally distributed data [35].

In the present application, FPCA extracts the principal modes of variations and allows examining if each subject’s *HGS*(*t*) mean curve (trials’ average) is a stable and reliable representation of their force pattern. This then ensures that any observed differences between groups (detected later by SnPM) are a consequence of genuine group differences and not of intra-subject variations.

On the other hand, SnPM performs continuous statistical hypothesis testing across the entire *HGS*(*t*) curve. It allows establishing specific time clusters where the curves are significantly different across groups, thus providing localized time-domain inference focused on the fundamental shape differences. Hence, it also strengthens the reliability and stability of cross-studies inference.

The study hypothesizes that such analysis will reveal temporal features and group differences that are not captured by conventional discrete measures such as maximum force and rates of force development. A further goal is to evaluate whether the *HGS*(*t*)-based approach can offer deeper insights into neuromuscular control and muscle function in older adults.

Ultimately, this framework could support the potential of the time-dependent *HGS*(*t*) curve as a very informative biomarker of functional capacity.

## 2. Materials and Methods

### 2.1. Participants and Screening

Participants were community-dwelling older adults recruited from a northern Portugal community area. Participants who were non-ambulatory, had any musculoskeletal problem that would prevent them from performing the *HGS*(*t*), or had a body mass index over 30 were excluded.

### 2.2. Experimental Protocol

The study followed a cross-sectional observational design. Handgrip strength recordings, expressed as *HGS*(*t*) curves, were obtained during a single testing session.

Participants performed the *HGS*(*t*) test with a digital handheld dynamometer, GripWise (Gripwise Tech, LDA, Braga, Portugal) [36], that is a fully instrumented IoT device with innovative mechanical features and wireless connectivity for real-time data acquisition. It utilizes Gripwise software (v1.0.15) for real-time data acquisition, s instant *HGS*(*t*) curve visualization and secure data storage in coded databases.

The *HGS*(*t*) test was conducted with participants sitting on a chair with back support. The shoulder was abducted and neutrally rotated, creating a 90-degree angle at the elbow joint. Participants executed 3 trials with their dominant hand. They were instructed to perform their maximal isometric strength for the total duration of 15 s, with 90 s intervals between trials. From the start of the test until the last second, they were encouraged verbally to exert and maintain their maximum force.

### 2.3. Data Analysis

#### 2.3.1. Data Preprocessing

The data were obtained using the GripWise device [36], with a rate of 100 samples/second during a 15 s handgrip strength test, yielding 1500 data points per curve. The data were preprocessed prior to applying FPCA and subsequent SnPM analyses.

Only subjects with three valid trials were considered. A 4th-order Butterworth filter (20 Hz, zero-lag) was applied to the *HGS*(*t*) curves to attenuate high-frequency noise while preserving the profile of voluntary force production. This is a commonly used practice in movement/force signal processing [37,38].

For the filtered curves, force onset, *Fonset*, was established as the earliest time point at which the force signal began a sustained rise (non-negative slope for 30 ms) that continued (within a small tolerance) until the force reached 90% of its peak value [17,39,40].

The curves were normalized to peak force, and the analysis was performed on both raw and normalized curves.

#### 2.3.2. Segmentation of the *HGS*(*t*) Curve

The force–time profile has different phases of force development; therefore, the FPCA + SPM pipeline was applied to the whole *HGS*(*t*) curve as well to the following time segments:

- *tonset* to 250 ms, to analyze the early explosive phase. This is one of the standard windows used for defining rates of force development, since it is dominated by neural drive, reaction time and initial muscle activation kinetics [5,17].
- *tonset* to *t63*, where *t63* represents the instant at which force first reaches 63.2% of its peak value. This characteristic response time interval was established under the assumption that the *HGS*(*t*) curve corresponds to the step response of a first-order system [41], thereby isolating the initial exponential rise phase.
- *t63* to *tmax* is the middle rising segment, capturing later recruitment and a different shape of the curve before maximum force is reached at *tmax*, complementing the early phase [41].
- *tmax* to *tfinal* is a segment that characterizes the post-peak behavior until the end of the test at *tfinal*. It can be associated with static fatigability [42,43].

**Figure 1:**
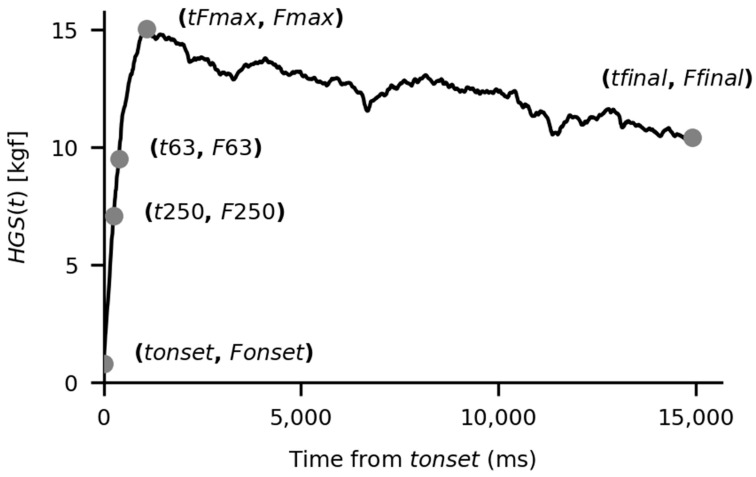
Time segmentation of a typical handgrip curve. Key instants are indentified from force onset: *tonset* (*Fonset*)*,* *t250* (*F250*), *t63* (*F63*), *tmax* (*Fmax*), *tfinal* (*Ffinal*) The dots separate the segments of the filtered *HGS*(*t*) for which FPCA + SPM was applied.

A typical HGS curve is depicted in Figure 1. The marked data points represent the boundary extremes for each time interval of the segmentation described.

Exploring the application of FPCA to these segments may reveal more than just what is obtained from the whole curve. Each sub-curve is less complex than the whole curve, which may lead to easier interpretations of FPCA for each phase.

Also, it will be possible to precisely detect, with SnPM, at which time clusters the curves differ.

#### 2.3.3. Functional Principal Component Analysis to Assess Intra-Trial Consistency and Validity of Using the Mean *HGS*(*t*) Curve

After pre-processing the data, functional principal component analysis (FPCA) was employed to verify that using the mean curve, obtained by averaging the three trials of each participant, provided a reliable representation of their individual *HGS*(*t*) performance.

FPCA was applied to the three curves of each trial (all recorded on the same 15 s, 100 Hz Grid) in two ways: raw force curves, and maximum force-normalized curves.

Raw FPCA preserves magnitude information, so components can reflect differences in absolute force. On the other hand, FPCA applied to normalized curves emphasizes shape and may yield components that are easier to interpret across individuals.

The validation metrics used to measure reliability, consistency, and variance explained, and which test the effectiveness of the FPCA model, are described in detail in Appendix A.1.

#### 2.3.4. Statistical Non-Parametric Mapping for Assessing Female/Male *HGS*(*t*) Differences

SnPM [35] was applied to different handgrip strength average curves. Specifically, SnPM analyses were conducted on the raw *HGS*(*t*) curves, the normalized curves, expressed as *HGS*(*t*)/*Fmax*, the first derivatives of the raw curves, and the first derivatives of the normalized curves. This approach aims at detection of amplitude, shape, and rate-related differences.

For each segment of the curves, the time axis was normalized to a unique unit interval (τ ∈ [0, 1]) to facilitate interpretation of cluster locations and to allow each analyzed segment to be expressed on a consistent percentage scale (0–100%).

The results of SnPM are presented for each time segment, both for the raw data and for the normalized to peak force curves. The information reported consists in the number of time clusters, the *p*-value for each cluster, the normalized times at which the cluster begins and ends, the cluster width, and the maximum value of t-statistics for each cluster.

Graphical representations of the results include male and female HGS functions plotted against normalized time. These are accompanied by SnPM{t} curves [27] where clusters indicating statistically significant differences between the two groups are explicitly highlighted.

Details of the statistical non-parametric mapping (SnPM) implementation are presented in Appendix A.2.

#### 2.3.5. Discrete Parameters Obtained from *HGS*(*t*) Curves

The discrete parameters chosen to describe relevant features of the *HGS*(*t*) curves, related to the time segmentation performed for the trial-averaged curves, are defined in Table 1.

Area 1 is the theoretical area under the HGS(t) curve, assuming Fmax remains constant from tmax up to tfinal (15 s). In other words, it equals Fmax × (tfinal − tmax). Area 2 is the actual calculated area under the HGS(t) curve from tmax to 15 s.

Comparison of these pre-specified descriptors across sexes were performed using the non-parametric Mann–Whitney U test with max|Z| adjustment to control for family-wise errors. Reported are the medians and interquartile ranges of each variable for female and male cases, the unadjusted and adjusted *p*-values, the Hodges–Lehmann estimator (HL) and the corresponding 95% confidence intervals.

## 3. Results

### 3.1. Participants

In this study, only participants with three valid trials were considered. The demographics are described in Table 2.

### 3.2. FPCA Results

Overall, the FPCA results indicate that the three trials per participant were very consistent in shape, both for normalized (Table 3) and for raw curves (Table 4). Across segments, the first principal component, PC1, typically explained the large majority of trial-to-trial variance, with only modest contributions from the second one, PC2. These occur especially in the t*Fmax*–15 s segment where force decays, and in the global curve. In fact, the shape of the whole curve is distinct from *tonset* to t*Fmax* and from t*Fmax* onward, which may account for the bigger contribution from PC2.

The dispersion of PC1 scores within participants (FPCA1_std) was small, and the within-participant RMSD was relatively low, both in absolute units and when expressed relative to individual peak force (RMSD_p), implying that differences between trials were small relative to the signal magnitude.

This characteristic, dominance by PC1 plus low within-subject dispersion supports the assumption that each participant’s trials shared a common underlying waveform with only small random variation. Therefore, averaging the three trials to obtain a single mean curve per participant was justified and provided a reliable representation for subsequent inferential analyses.

### 3.3. SPM Results

Statistical parametric mapping identified clear differences in the temporal structure of the handgrip force–time curves between women and men. When force was normalized to each participant’s maximum (curve type Norm in Table 5), differences were confined to the very early portion of the curve. In the *tonset*–*250 ms* and *tonset*–*t63* intervals, normalized force was slightly higher in women, characterized by significant brief clusters.

**Figure 2:**
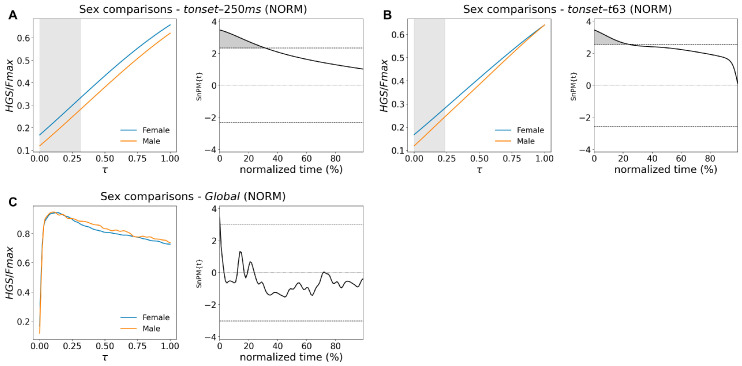
Female–male SnPM comparison of *HGS* normalized curves across three segments: *tonset*-250 ms (**A**), *tonset-t63* (**B**), and *t63-tmax* (**C**). **Left panels** show mean curves (female: blue, male: orange) vs. normalized time, τ. **Right panels** show the corresponding SnPM{t} curves. Gray clusters indicate significantly higher female normalized *HGS* values. Brief clusters occur during early phases of *HGS*(*t*)/*Fmax*, whereas for the *Global* segment, the two curves overlap.

As illustrated in Figure 2A,B, the left panels display the mean trajectories for both sexes across normalized time (τ ∈ [0, 1]; 0 segment start, 1 segment end), while the right panels show the corresponding curves as a function of normalized time expressed in percentage. These right-hand plots highlight the specific temporal windows where the SnPM{t} values exceed the significance threshold.

No differences were detected in the subsequent rising or decaying portions of the normalized curves. Figure 2C shows the results for the full curve, where no differences between men and women were detected.

**Figure 3:**
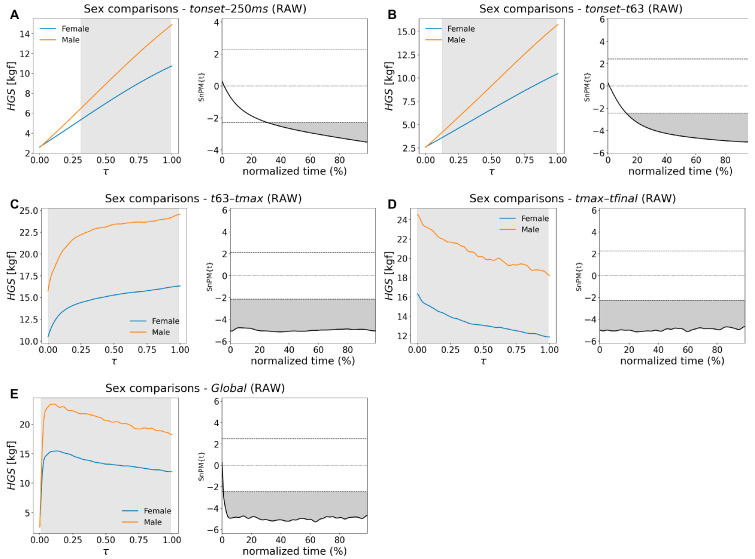
Female–male SnPM comparisons of *HGS*(*t*) curves across all segments: *tonset*-250 ms (**A**), *tonset*-*t63* (**B**), *t63*-*tmax* (**C**), *tmax*-*tfinal* (**D**), and Global (**E**). (**Left panels**) show mean curves (female: blue, male: orange) as a function of normalized time, τ. (**Right panels**) show the corresponding SnPM{t} curves. Significant clusters (highlighted in gray) were identified across all segments, indicating that male values were significantly higher than female ones.

The analysis of raw force curves produced a different pattern (curve type Raw in Table 5). Across nearly all phases, men generated higher absolute force than women, with differences emerging shortly after movement onset. Significant regions were consistently identified, as depicted in Figure 3: from *tonset* to 250 ms (Figure 3A); from *Fonset* through the rise to 63.2% of peak force (Figure 3B), from *t63* to *tmax* (Figure 3C), and throughout the force-decay period (Figure 3D). When the curve was examined as a whole, male force values remained significantly above female values for nearly the entire time course (Figure 3E).

**Figure 4:**
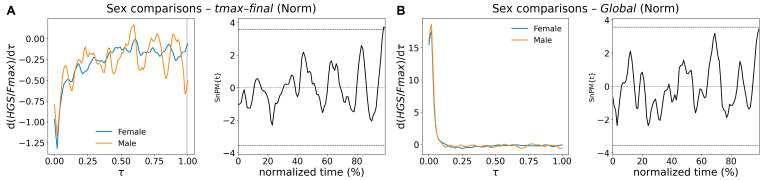
Female–male SnPM comparisons of derivatives of HGS normalized curves across segments *tmax-tfinal* (**A**) and *Global* segment (**B**). (**Left panels**) show mean curves (female: blue, male: orange) as a function of normalized time, τ. On the (**right**), the corresponding SnPM{t} curve against the same normalized time (in percentage). A very brief significant cluster, highlighted in gray, was detected in (**A**).

For normalized derivatives (curve type Norm of Table 5), the only significant difference was detected at the very end of the decay phase segment (*tmax*-*tfinal*), where the threshold is barely surpassed and the cluster is very short (Figure 4A). For all the other segments, the SPM{t} trajectories remained below the critical threshold. Figure 4B illustrates the superposition of the normalized derivative curves for both female and male groups and the respective SnPM{t} curve that never exceeded the threshold value.

**Figure 5:**
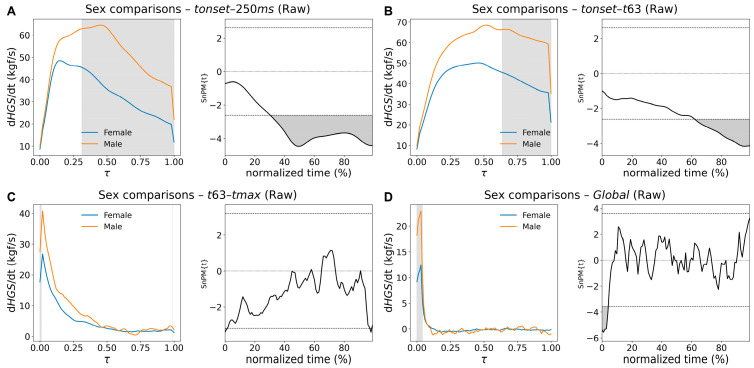
Female–male SnPM comparisons of derivatives of HGS raw curves across segments *tonset-t250 ms* (**A**), *tonset-t63* (**B**), *t63-tmax* (**C**), and *Global* (**D**). **Left panels** show mean curves (female: blue, male: orange) as a function of normalized time, τ. On the **right**, the corresponding SnPM{t} curve against the same normalized time (in percentage). Significant clusters were detected in (**A**,**B**) and at the early phase in (**D**). There are two very short clusters at the beginning and end of (**C**).

In contrast, analysis of raw derivatives revealed several regions of significant sex-related differences during the rising phase (Table 6, curve type Raw and Figure 5).

In the *tonset*–250 ms interval (Figure 5A), a long cluster covered most of the segment, indicating higher rates of force increase in men. A second cluster appeared near the end of the *tonset*–*t63* interval, again showing higher derivative values in men. Near the peak-force phase (*t63*–*tmax*), the curves were largely overlapping, with two brief significant clusters at the segment’s beginning and end.

No differences were observed in the decay phase (*tmax*−*tfinal*). When the full derivative curve was examined, only a short initial interval reached significance, indicating that differences in the rate of force development were concentrated in the early phase of *HGS*(*t*).

### 3.4. Results for Sex Differences in HGS Descriptors

Table 7 presents the non-parametric comparisons of discrete *HGS*(*t*) parameters between male and female participants. As expected, absolute forces are all greater in men: *Fmax*, *F63* and *Ffinal*. The 95% CIs are large, which likely reflects the unbalanced sample sizes, or high within-group variability. In fact, the IQRs of male force distributions are larger than those of women. Timing measures, *tmax* and *t63*, do not differ, consistent with the findings of reference [41].

As to the rates of force development, the early rise in force, captured by *RFD*250, is higher in men; post peak decay, assessed by *RFD*3, is also greater in absolute value for men than for women, meaning that men reach higher maximum force, but it decays faster, a feature already reported in [41]. Here too, the 95% confidence intervals of the Hodges–Lehmann estimators are relatively wide, indicating considerable uncertainty about the true magnitude of the effect.

The mid-phase rates described by *RFD*1 and *RFD*2 are not different, reinforcing that the divergence is localized at the initial force rise. On the other hand, the fatigability index, a normalized quantity, is also not different for men and women.

**Figure 6:**
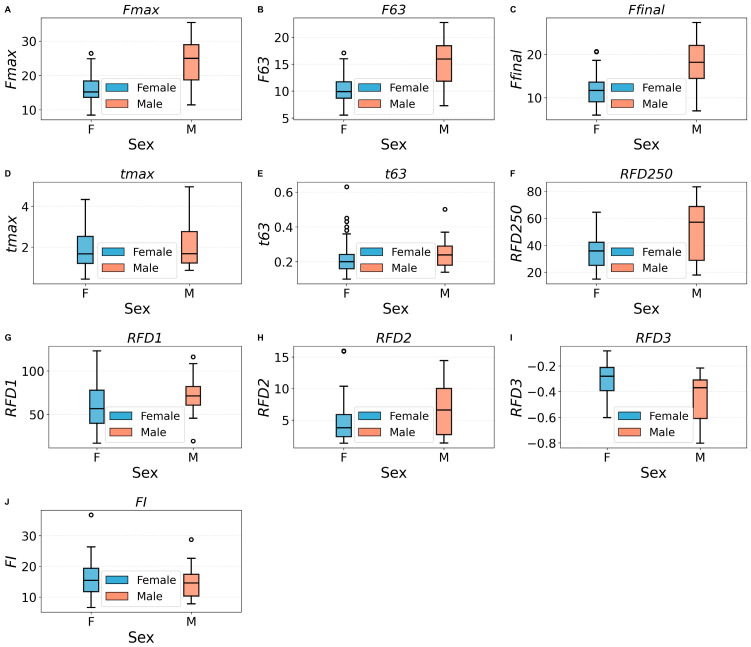
Boxplots illustrating sex differences in the distributions of discrete parameters. Significant differences were detected for the force variables (plots (**A**–**C**)), early-phase RFD (plot (**F**)) and in decay rate (plot (**I**)). Time descriptors show similar distributions across sexes (plots (**D**,**E**)), as well as RFD1 (plot (**G**)), RFD2 (plot (**H**)), and the fatigability index I (plot (**J**)).

The boxplots in Figure 6 illustrate the results presented in Table 7.

## 4. Discussion

Time-normalized *HGS*(*t*) curves and their derivatives were analyzed using a functional pipeline combining FPCA and SnPM. As far as the authors know, this methodology is novel in the context of time-profile handgrip strength studies. This approach allows identification of localized clusters of sex-related differences across the normalized time domain and confirms that trial-averaged curves provide a reliable individual representation of *HGS*(*t*), supporting their use in group-level comparisons.

FPCA + SnPM results partially agreed with sex differences in discrete force, time, and force-development estimators. For raw curves, SnPM showed that men’s handgrip strength is significantly higher, which aligns with existing observations of the discrete descriptors [44,45]. Differences in the derivatives of raw curves at the end of the *tonset*–*250 ms* segment align with the differences observed in the discrete *RFD*250 *ms*. Furthermore, the early-phase divergency in the *Global* segment is consistent with the fact that aged males retain a higher proportion of type II fibers compared with women, allowing for greater force and *RFD* generation [46,47].

In the *tonset*–*t63* segment, SnPM identified a late cluster (60–100% of the segment), while *RFD1* did not differ between sexes. This likely occurs because the discrete measure averages the entire segment, diluting localized late differences so that group medians become non-significant even when a late cluster is present. In contrast, SnPM found no clusters between *tmax* and *tfinal*, whereas *RFD*3 suggested a faster decline in men. This discrepancy may stem from the oscillatory nature of the force signal during decay, where excursions above significance thresholds may be too brief for cluster-wise error control, yet still shift the segment-level average. Furthermore, the higher proportion of type I fibers in women may allow for a greater fatigue resistance, which may explain, at least in part, the faster decline observed among males [47].

The ability of SnPM to detect localized differences where no corresponding discrete *RFD* differences were observed suggests that the functional approach can reveal temporal features and group differences not captured by discrete parameters. Conversely, for the decay phase, SnPM detected no clusters even though *RFD3* differed between sexes, likely due to the rapid oscillatory nature of the signal.

A relevant strength of this method is that it enables distinguishing differences that occur due only to amplitude from those occurring due to shape or timing. The present results indicate that sex differences in older adults’ handgrip performance are primarily amplitude (capacity) related, reflecting greater overall force capacity in men rather than differences in the shape or timing of the force–time profile. In absolute terms, males exhibited higher forces and greater very-early rates of force development (0–250 ms), with SnPM identifying significant differences in the early phases of *HGS*(*t*) derivatives. After normalization, however, FPCA + SPM revealed no shape or timing-specific clusters, reinforcing the interpretation that differences result from different strength capacities, not from distinct neuromuscular control strategies, which can be explained, at least in part, by the reported gender differences in muscle fiber type distribution [47]. Nonetheless, one should be mindful of normalizing force data to maximal force, as this may exacerbate early-phase force increases among those with lower peak force, which may be applicable when comparing women and men [17,48].

The main limitation of the present study is the fact that the sample is relatively small and very unbalanced. The strict inclusion criteria requiring three valid *HGS*(*t*) trials per participant limited the final sample, especially for the male group. Consequently, there was an increased risk of not detecting genuine physiological differences due to reduced statistical power (false negatives). However, the use of the non-parametric permutation-based approach (SnPM) offered the advantage of providing a robust statistical threshold that does not rely on the assumption of normality. Therefore, the influence of outliers was mitigated, avoiding the risk of false positives.

Many extensions and improvements can be made in similar analyses, such as performing other normalizations of force (dividing the curves by body mass or by forearm circumference), calculating rates of force development that match the phases of *HGS*(*t*) corresponding to the clusters obtained in SnPM to provide scalar summaries aligned with the functional signal, and considering *HGS*(*t*) tests of 30 s or more to better assess the decay segment of the curves.

Future analysis could extend the application of the FPCA + SPM framework to explore differences in *HGS*(*t*) across broader populations and contexts. For instance, comparing time force profiles between younger and older adults could provide understanding on age-related changes in neuromuscular performance. Similarly, analysis involving individuals with different levels of physical fitness, or those affected by specific musculoskeletal conditions, may reveal distinct patterns of strength production and maintenance. By comparing how the *HGS*(*t*) curve evolves under different physiological or pathological conditions, such approaches could contribute to a deeper understanding of muscle function and provide more sensitive information than discrete measures alone.

Furthermore, these findings may have substantial implications for exercise prescription and physical practice for healthy older and clinical populations. Specifically, exercise interventions may be tailored to attenuate certain deficits identified in the neuromuscular force profiles. Considering that deviations from “healthy” force generation patterns may be indicative of underlying neuromuscular impairments, this approach may also be a valuable tool in clinical practice to help identify pathological conditions.

## 5. Conclusions

This study demonstrates that, for older adults, sex-based differences in the handgrip strength time profile are primarily driven by magnitude rather than by distinct muscle control strategies.

By employing a functional pipeline (FPCA + SPM), it was established that the average of the three trials provides a reliable representation of the individual *HGS*(*t*) curves, capturing consistent variability that discrete measures may overlook.

Furthermore, the identification of specific temporal phases where raw and normalized curves diverge confirms that elderly women and men utilize similar muscle control mechanisms despite differences in absolute force.

The major limitation is the small and unbalanced sample size that impacts the statistical power of the analysis and may limit the generalizability of the findings. Therefore, further studies with larger sample sizes and different cohorts could provide a more robust validation of the methodology and determine if the observed temporal differences in *HGS*(*t*) curves remain consistent across broader populations.

## Figures and Tables

**Table 1:** Definition of the discrete parameters calculated using the trial-averaged *HGS*(*t*) curves. Indicated are the symbols, descriptions of the parameters, units used, and the formulas defining some parameters as a function of others.

Symbol	Descriptor	Units	Formula
*Fmax*	Peak force	kgf	
*tmax*	Time to peak force	s	
*F63*	Force at 63.2% of peak value	kgf	
*t63*	Time to 63.2% of peak force	s	
*Ffinal*	Force at end of test (15 s)	kgf	
*RFD*250	Rate of force development (0–250 ms)	kgf/s	(*F*(250 ms) − *Fonset*)/0.250
*RFD*1	*RFD* from *Fonset* to *F63*.2	kgf/s	(*F63* − *Fonset*)/(*t63* − *tonset*)
*RFD*2	*RFD* from *F63*.2 to *Fmax*	kgf/s	(*Fmax* − *F63*)/(*tmax* − *t63*)
*RFD*3	Decay rate from *Fmax* to end of test	kgf/s	(*Ffinal* − *Fmax*)/(*tfinal* − *tmax*)
*FI*	Static fatigability index	-	((*Area*_1_ − *Area*_2_)/*Area*_1_ × 100

**Table 2:** Participant demographics.

	Female	Male
N	44	13
Age, years	71.4 ± 5.8	72.9 ± 4.5
Height, cm	156.2 ± 5.7	167 ± 5.5
Weight, kg	65.6 ± 5.7	75.3 ± 8.9
BMI, kg/m^2^	26.8 ± 2.5	27.0 ± 2.9

**Table 3:** Results for relevant FPCA measures obtained for each time segment of the normalized curves by sex group. PC#_var is the median of the percentage variance explained by the two components. RSMD is the average of within-participant root mean square deviation, and RSMDp is the normalized RSMD expressed as a percentage of maximum force.

Segment	Sex	FPCA1_std	FPCA2_std	RMSD	RMSDp	PC1_var (%)	PC2_var (%)
*tonset*–*250 ms*	F	0.807	0.114	0.0758	7.58	98.3	1.73
*tonset*–*250 ms*	M	0.883	0.118	0.0831	8.31	98.0	1.97
*tonset*–*t63*	F	0.285	0.068	0.0276	2.76	95.2	4.79
*tonset*–*t63*	M	0.393	0.105	0.0384	3.84	95.3	4.72
*t63*–*tmax*	F	0.294	0.121	0.0309	3.09	83.1	16.9
*t63*–*tmax*	M	0.282	0.108	0.0299	2.99	85.2	14.8
*tmax*–*tfinal*	F	0.406	0.201	0.0446	4.46	79.4	20.6
*tmax*–*tfinal*	M	0.401	0.245	0.0466	4.66	69.3	30.7
*Global*	F	0.440	0.239	0.0494	4.94	75.5	24.5
*Global*	M	0.420	0.271	0.0496	4.96	68.4	31.6

**Table 4:** Results for relevant FPCA measures obtained for each time segment of the raw curves by sex group. PC#_var is the median of the percentage variance explained by the two components. RSMD is the average of within-participant root mean square deviation, and RSMDp is the normalized RSMD expressed as a percentage of maximum force.

Segment	Sex	FPCA1_std	FPCA2_std	RMSD	RMSDp	PC1_var (%)	PC2_var (%)
*tonset*–*250 ms*	F	12.9	1.87	1.22	5.53	98.1	1.90
*tonset*–*250 ms*	M	22.2	3.16	2.10	8.67	97.2	2.80
*tonset*–*t63*	F	5.20	1.74	0.527	2.41	92.7	7.30
*tonset*–*t63*	M	11.1	3.24	1.10	4.59	91.0	9.00
*t63*–*tmax*	F	9.26	2.72	0.923	2.42	92.4	7.60
*t63*–*tmax*	M	12.5	3.44	1.25	3.50	90.3	9.70
*tmax*–*tfinal*	F	8.43	3.58	0.890	1.74	85.6	14.4
*tmax*–*tfinal*	M	13.9	6.33	1.50	3.88	78.4	21.6
*Global*	F	8.91	4.40	0.974	1.82	80.2	19.8
*Global*	M	14.2	6.87	1.55	3.80	74.4	25.6

**Table 5:** Summary of statistical parametric mapping results for each segment of HGS curve expressed in normalized time. For each curve type, raw (Raw) and normalized (Norm), there is indication of the number of clusters (nc), the *p*-values, cluster start and end points (% of normalized time), cluster extent and maximum absolute t-value (max|t|).

Segment	Curve Type	nc	*p*-Value	Cluster Start (%)	Cluster End (%)	Cluster Extent	Max|t|
*tonset*–*250 ms*	Raw	1	0.0052	55.5	100	44.5	3.54
*tonset*–*t63*	Raw	2	0.0004	19.1	100	80.9	5.35
*t63*–t*Fmax*	Raw	1	0.0004	0	100	100	5.08
*tmax*–*tfinal*	Raw	1	0.0004	0	100	100	5.26
*Global*	Raw	1	0.0004	1.10	99	97.9	5.28
*tonset*–*250 ms*	Norm	1	0.0152	0	27.3	27.3	3.47
*tonset*–*t63*	Norm	1	0.0192	0	25.8	24,8	3.68
*t63*–t*Fmax*	Norm	0	-	-	-	-	-
*tmax*–*tfinal*	Norm	0	-	-	-	-	-
*Global*	Norm	0	-	-	-	-	-

**Table 6:** Summary of statistical parametric mapping results for each segment of the derivative of HGS curves expressed in normalized time. For each curve type, raw (Raw) and normalized (Norm), there is indication of the number of clusters (nc), the *p*-values, cluster start and end points (% of normalized time), cluster extent and maximum absolute t-value, (max|t|).

Segment	Curve Type	n_c_	*p*-Value	Cluster Start (%)	Cluster End (%)	Cluster Extent	Max_|t|_
*tonset*–*250 ms*	Raw	1	0.0004	30.3	100	69.7	4.48
*tonset*–*t63*	Raw	1	0.0004	61.6	100	38.4	4.18
*t63*–t*Fmax*	Raw	2	0.0116 0.0116	097.0	2.00 99.0	2.00 2.00	3.41
*tmax*–*tfinal*	Raw	0	-	-	-	-	-
*Global*	Raw	1	0.0004	1.10	100	98.9	5.28
*tonset*–*250 ms*	Norm	0	-	-	-	-	-
*tonset*–t*F63*.2	Norm	0	-	-	-	-	-
t*F63*.2–t*Fmax*	Norm	0	-	-	-	-	-
t*Fmax*–*tfinal*	Norm	1	0.048	98.2	100	1.80	3.72
*Global*	Norm	0	-	-	-	-	-

**Table 7:** Discrete parameter statistics and sex comparison test results. Indicated are the medians and respective interquartile ranges (IQRs), the observed statistics (stat), unadjusted (*p*) and adjusted (*p*-adj) *p* values, the HL estimator and respective 95% confidence intervals (CIs).

Descriptor	Female Median [IQR]	Male Median [IQR]	Observed Stat	*p*	*p*-adj	HL	95% CI-HL
*Fmax* (kgf)	15.2 [13.6–18.4]	25.0 [18.7, 28.9]	−0.584	0.001	0.007	−8.97	[−13.6, −2.58]
*tmax* (s)	1.67 [1.19–2.52]	1.68 [1.21, 2.76]	−0.094	0.612	0.994	−0.12	[−0.91, −0.42]
*F63* (kgf)	9.89 [8.65–11.7]	15.9 [11.8, 18.4]	−0.573	0.002	0.008	−5.76	[−8.80, 1.55]
*t63* (s)	0.20 [0.160–0.242]	0.24 [0.18, 0.29]	−0.203	0.274	0.802	−0.02	[−0.08, 0.02]
*Ffinal* (kgf)	11.7 [9.07–13.6]	18.2 [14.4, 22.0]	−0.566	0.002	0.010	−6.52	[−10.3, −2.21]
*RFD*250 (kgf/s)	35.9 [25.0–42.25]	57.1 [28.8, 68.7]	−0.503	0.005	0.035	−19.3	[−31.7, −3.03]
*RFD*1 (kgf/s)	56.6 [39.7–77.6]	71.2 [60.8, 81.9]	−0.318	0.082	0.385	−16.0	[−31.3, 2.09]
*RFD*2 (kgf/s)	3.79 [2.37–5.88]	6.60 [2.71, 10.0]	−0.203	0.271	0.802	−1.33	[−5.29, −0.89]
*RFD*3 (kgf/s)	−0.280 [−0.392–−0.211]	−0.37 [−0.61, −0.31]	−0.489	0.006	0.043	0.14	[0.036, 0.31]
FI	15.4 [11.8–19.3]	14.6 [10.3, 17.4]	0.140	0.455	0.953	1.19	[−2.19, 4.02]

## Data Availability

The datasets used and analyzed in the present study are available from the corresponding author on reasonable request.

## Abbreviations

The following abbreviations are used in this manuscript:

HGS	Handgrip Strength
FPCA	Functional Principal Component Analysis
SPM	Statistical Parametric Mapping
SnPM	Statistical non-Parametric Mapping
RFD	Rate of Force Development
pRFD	Normalized Rate of Force Development
RMSD	Root Mean Square Deviation
PC	Principal Component

## Appendix A

### Appendix A.1. Functional Principal Component Analysis (FPCA)

After pre-processing the data, FPCA was applied to validate the use of each participant’s mean curve as a representative summary of their *HGS*(*t*) trials.

The metrics described below quantify how closely each trial matches the participant’s mean curve (reliability), how well FPCA summarizes the curve variability, and how stable the extracted patterns are across trials (effectiveness of the model).

FPCA Reliability Metrics (Trial Consistency)

- Root mean square deviation (RSMD)—Measures the average deviation of each trial from the participant’s mean curve. Lower values indicate higher intra-individual consistency across trials.
- Normalized RMSD (RMSDp)—RMSD expressed as a percentage of participant’s peak force allows comparison of reliability between individuals with different absolute strength levels. A low percentage indicates stable trial-to-trial behavior regardless of strength.

FPCA Effectiveness Metrics (Model Dimensionality and stability)

- Percentage of variance explained (PC#_var)—Median proportion of total curve variability explained by each principal component. High values indicate the most individual differences in *HGS*(*t*) can be captured by a small number of principal components, which asserts effective dimensionality reduction.
- Standard deviations of FPCA scores (FPCA_#sd)—Average standard deviation of principal component scores across the three trials. Low values indicate that each participant’s trial scores similarly on the principal components, confirming stable curve shape and reliable pattern extraction.

Statistical analysis and data processing were performed using Python (3.11.13). Data manipulation was handled via Pandas (2.2.3) and NumPy (2.3.4). Principal Component Analysis (PCA) and error metrics were implemented using Scikit-learn (1.7.0). Linear modeling and Analysis of Variance (ANOVA) were conducted using Statsmodels (0.14.4), with SciPy (1.15.3) utilized for probability distribution calculations and *p*-value extraction.

### Appendix A.2. Statistical Non-Parametric Mapping

For time segments reduced to 100 data points, derivatives were computed by a centered finite difference, then smoothed by a 5-point moving average. When differentiating raw force on the normalized time axis τ, units are kgf·$\tau^{-1}$; to express physical rates, the conversion used is dF/dt=(1/duration) dF/dτ (kgf·s^−1^).

In contrast to the parametric SPM, the non-parametric permutation version makes no assumptions about the specific distribution of the data.

The null hypothesis assumes that the sex label assigned to the participant (female or male) has no effect on the resulting time curve. If this is true, then the difference between the group averages is merely random variation. To test this, the method takes all individual time curves and randomly mixes up their group labels. It then recalculates the difference between the two new, randomly formed groups.

In the present calculation, 5000 thousand iterations were used, i.e., the sex labels were shuffled 5000 thousand times, each time resulting in a new, random statistical map, which is a curve representing the comparison test statistics (t-value) as a function of normalized time. From the maximum absolute t-value of each permuted curve, the test builds an empirical null distribution, which is a comprehensive picture of the differences that would happen purely by chance. This null distribution is then compared to the one obtained from the actual correctly labeled data. If the result from the actual data is more extreme than 95% percent of the 5000 random results, it will be considered statistically significant (significance level of 0.05). A two-tailed comparison test was applied, meaning that both possibilities were considered for the differences (Female > Male and Male > Female).

SnPM was conducted using spm1d (0.4.52). All functional data visualizations were generated using Matplotlib (3.10.0).
